# A Conceptual Framework for the Social Analysis of Reproductive Health

**Published:** 2007-03

**Authors:** Neil L. Price, Kirstan Hawkins

**Affiliations:** ^1^ Centre for Development Studies, University of Wales Swansea, Singleton Park, Swansea, SA2 8PP, UK; ^2^ PEER Unit, Options Consultancy Services, CAP House, 9–12 Long Lane, London EC1A 9HA, UK

**Keywords:** Social analysis, Social appraisal, Reproductive health, Human immunodeficiency virus, AIDS

## Abstract

The dominant conceptual framework for understanding reproductive behaviour is highly individualistic. In this article, it is demonstrated that such a conceptualization is flawed, as behaviour is shaped by social relations and institutions. Using ethnographic evidence, the value of a social analysis of the local contexts of reproductive health is highlighted. A framework is set out for conducting such a social analysis, which is capable of generating data necessary to allow health programmes to assess the appropriate means of improving the responsiveness of service-delivery structures to the needs of the most vulnerable. Six key issues are identified in the framework for the analysis of social vulnerability to poor reproductive health outcomes. The key issues are: poverty and livelihood strategies, gender, health-seeking behaviour, reproductive behaviour, and access to services. The article concludes by briefly identifying the key interventions and strategies indicated by such an analysis.

## INTRODUCTION

Reproductive behaviour is embedded within specific social relations and political and cultural contexts. Creating conditions which support behaviour change—a critical dimension of health and HIV/AIDS policy and programme development—requires analysis of these contexts. However, the dominant conceptual framework for understanding reproductive behaviour is highly individualistic, derived from the fertility cost-benefit models espoused by Becker (1) and Easterlin (2), with the unit of decision-making being the individual or the ‘reproductive’ couple (3, 4—for useful summaries of cost-benefit models in fertility theory). Ethnographies have demonstrated that such a paradigm is flawed both in its understanding of human action and in its assumptions about the central units of reproductive decision-making. Far from being an individual decision-making process, reproductive behaviour is shaped by social relations and institutions at the local level, such as kinship groups, informal social networks, local political institutions, and religious and spiritual advisors and healers, which are influenced by and the product of the wider social, political, economic and historical processes (5–11).

Inattention to context is evident in many reproductive health interventions and policies. For example, the reproductive rights discourse focuses on the rights of the individual, often to the exclusion of the wider social and economic conditions within which rights are defined and realized. Such a perspective underplays the extent to which the poor and vulnerable are unable to realize their rights to the economic and social resources vital for the protection of their health and well-being.

In this article, we demonstrate the need for social analysis to generate an understanding of the diverse contexts of reproductive health, the ways in which needs and priorities are identified, especially among marginalized groups* and addressed (through *inter alia* health-service provision) and the social dynamics of exclusion and vulnerability. We start with ethnographic illustrations of how sociocultural, economic and political factors shape reproductive behaviour in relation to four key areas: fertility, culture, gender, and sexuality. We limit our discussion to these four themes because of space limitations, and, in part, because of our professional expertise. However, we acknowledge the impact of wider factors on reproductive behaviour, such as education, access to healthcare, occupation, marital status, and harmful traditional practices.

Following the discussion of context, we set out a framework for conducting a social analysis. Data generated by such a social analysis will enable programmes to assess appropriate means of improving the responsiveness of service-delivery structures, including the quality of care they provide. The article concludes by briefly identifying key interventions and strategies indicated by such an analysis.

## THE SOCIAL CONTEXT OF REPRODUCTIVE HEALTH

### Fertility

Most family-planning programmes and fertility-control policies have traditionally failed to take adequate cognisance of the complex forces influencing the demand for children.

In contexts of extreme poverty, for example, lack of resources to meet the rising cost of children are often taken to indicate a decline in demand for children, despite evidence that, in such contexts, children are valued as a source of social, economic and political security. The outcome under such conditions may not be increased demand for modern contraceptive services, but changes in the contexts in which children are conceived and in which they grow up. Increased poverty in many parts of the world combined with globalization of capital provide the context for increased entry of children into the workforce (as an economic resource to their families and as a cheap source of labour (12)), and into economically-based sexual relations (13–17).

Furthermore, children often have an essential symbolic value and are an important source of social support (9–11). Ancestral religion in many societies in sub-Saharan Africa, for example, ascribes an indispensable role to children in the maintenance of the lineage, which is of central importance in the social and political organization of many such societies (9, 10). In China, the symbolic importance of children is translated into resistance to permanent methods of fertility control. The lineage is perpetuated by economic production and social reproduction, and consequently, the social worth of a person depends on the ability to work and to carry on the family line. Sterilization is seen as damaging the lineage, production, and reproduction and is viewed with more hostility than other methods of fertility control, including abortion (11).

### Culture

Within the mainstream reproductive health literature, the understanding of the role of culture in influencing behaviour has been largely informed by structural-functionalist social theory. Within this paradigm, typified by work such as that of Freedman (18), culture is (mis)understood as a set of prescribed norms that guide social behaviour, and attitudes are seen as synonymous with these cultural norms and expectations (18). Diffusion theory (19, 20), a dominant framework within reproductive health and underpinned by structural-functionalist normative theory, holds that the most important source of behaviour change is the spread of new ideas:

“… the process of modernisation or Westernisation can act as the source of ideational change affecting fertility behaviour through complex but undirected interactions. By contrast, ….[family planning] programs consciously direct contraceptive information, motivation, and services at specific populations through personal or impersonal communication. New ideas, knowledge, and practices are then spread further through informal social networks that include family members and peers” (21).

According to the diffusionist perspective, traditional culture is a barrier to behaviour change, with a great deal of research effort directed at identifying cultural barriers to contraceptive use (22–24). A similar emphasis on culture as barrier is evident in the literature on maternal health. Lack of education and the perpetuation of ‘false beliefs’ reinforced by traditional birth attendants are cited as major obstacles to improved maternal health (25).

Empirical and descriptive accounts of culture as norms tell us little about how, when, and why people choose to use norms to legitimize behaviour or when and why they adopt strategies which challenge taboos and contradict social norms. Lockwood highlighted this in his analysis of postpartum abstinence in West Africa, which showed clearly that “…there is no hegemonic postpartum norm, and men and women of different statuses and ages draw on a variety of normative statements to evade, promote, or undermine abstinence” (7). Most analyses of postpartum abstinence focus on taboos and social norms governing reproductive behaviour. Lockwood's analysis indicates that reproductive behaviour is negotiated within *competing* norms and taboos, such as between gender norms of sexuality, which pressure women to resume early sexual contact following childbirth and taboos on sex during lactation (7).

There is now a substantial body of literature which refutes the structural-functionalist view that behaviour is governed by social and cultural norms (5–7, 9–12). Culture is instead seen as a dynamic response to specific local circumstances: continuously created and recreated in the course of social interaction (6). This conceptualization of culture provides a lens through which to understand reproductive health decision-making. Rutenberg and Watkins, for example, showed how decisions to use family planning were not one-off events, but represented a continual process of negotiation and strategizing within social networks:

“Decisions appear to be preceded by a period during which women overhear or participate in conversations with others, and then by more strategic conversations when women seek out those whom they believe are using contraceptives. Once a woman begins to practice contraception, she continues with these conversations and she monitors her body's reaction, ready to discontinue use should she learn something disturbing about the experience of others or if the method does not ‘rhyme’ with her body” (26).

### Gender

Several studies have highlighted the need to understand reproductive behaviour in the context of the social construction of gender and sexuality (27–31). For example, a recent review article provides extensive ethnographic illustrations of influences of men on the reproductive health of women, in the areas of contraception, sexually transmitted infections, pregnancy and childbirth, infertility, and foetal harm (32). However, the majority of studies concerned with the relationships among gender, sexuality, and fertility have focused narrowly on identifying factors that would promote contraceptive acceptance and use-effectiveness to achieve more a rapid decline in fertility (28). Typically, these studies have relied upon evidence from survey research on the relationship between contraceptive method-choice and sexual experience and satisfaction. Dixon-Mueller argues that these subjective aspects of individual responses to the impact of contraceptive use on sexual pleasure are themselves mediated by broader social contexts, such as gender and class relations (28).

Even where local culture and gender relations support fertility regulation, the use of apparently accessible reproductive health services may remain extremely low (26, 33). Such low use-rates often do not reflect low demand for healthcare, but imbalances in power relations between health services and the community resulting in a rejection of services by certain groups (34). Local healers may continue to be used over and above biomedical health services, as a consequence of the significance of the social relations implicit in the provider-client encounter. A study among rural women in Kenya illustrates the point. “The women in the study area are ambivalent about family planning providers. They see them as the crucial sources of the complicated technical information they need to use these methods correctly. Yet the providers are socially distant from these rural women, who are unsure how to trust them. As a result women go back and forth between family planning providers and women whose bodies and circumstances are more like their own—such as the cleaners in the clinic…. We suspect that an important source of the provider's attitudes is their identification with the modern health sector. An aspect of the general view of modernity in the Western world is that its development is explained, at least partly, by ‘coming to see’ the value of scientific rationality and thus by the shedding of harmful myths.… Those engaged in the national family planning program see themselves as modern, by virtue of their education, medical training, and location in a modern institution. That they are dismissive of the information women have gleaned from their untrained friends and of what they regard as ‘myths and rumours’ is not surprising” (26).

Where social and political marginalization and poverty act as significant constraints on access to healthcare, women often continue to exploit localized strategies for fertility regulation, such as sexual abstinence. Ethnographic research among the Yoruba of Nigeria reveals that the possibility provided by modern contraceptives to divorce sexuality from reproduction is not universally perceived as a source of women's empowerment, with terminal sexual abstinence to end childbearing being viewed as a well-earned rest, such that the practice “…conflicts with the western liberal view of female sexual rights and the biomedical perception of biological needs” (35).

A study in the Gambia has shown how reproductive behaviour and decision-making, based on local understandings of bodily processes, “…fly in the face of every major demographic theory that has been advanced to explain fertility behaviors in Africa” (36). The use of contraceptives following reproductive mishaps, such as miscarriage, in a society that places a high value on fertility, does not correspond with conventional understandings of demand and supply. “[R]ural Gambians see fertility as limited by a woman's eroding bodily capacity to bear a child safely over successive pregnancy outcomes. This capacity wears out less with the passage of time than with the cumulative effects of wear and tear on the body, particularly in the wake of obstetric traumas. Since the pace of this decline can be slowed with ‘rest’ between pregnancies (that is, the creation of recuperative space), and since time spent in ‘resting’ is considered largely irrelevant to ultimate child numbers, it is not surprising that the most traumatic health assaults, such as those that reproductive mishaps reflect or intensify, produce the strongest contraceptive responses” (36).

In the rural Gambian context of high levels of reproductive morbidity and mortality, it is a health model, not a demographic one, which dominates people's thinking and decision-making about contraception and patterns of contraceptive use. Other anthropological studies have shown that knowledge and behaviour around fertility control are congruent with local health-belief systems. Rylko-Bauer showed how inducing menstruation ensures a regular flow of bodily substances, essential for the maintenance of health, and concluded that such decisions and practices are made in the context of gendered economic and kinship relations (31).

### Sexuality and HIV

There is an increasing body of literature emanating from HIV and AIDS research which demonstrates that sexual behaviour cannot be understood without reference to the social context in which it takes place (37–39). HIV and AIDS research during the 1980s was dominated by conceptual models aimed at explaining sexual attitudes and behaviours quantitatively. Psychological approaches emerged as the dominant conceptual framework, emphasizing individual as opposed to societal determinants of sexual behaviour. These approaches have been criticized for their failure to take account of the social relations and conditions that both constrain and give meaning to sexual behaviour. Parker, for instance, stated “Baseline behavioural data were collected, such as numbers of sexual partners, criteria for partner selection, the prevalence of condom use, and attitudes towards HIV infection and AIDS… However, these surveys provided little insight into many issues associated with effective intervention. One of the most consistent findings in many knowledge, attitudes, practices and beliefs (KAPB) surveys has been the limited impact knowledge of HIV infection seems to have on risk behaviour” (40).

Public-health interventions, which have been aimed at reducing so-called high-risk sexual behaviour, have often failed to address adequately the importance of social vulnerability, marginalization, and relations of power and control in influencing sexual behaviour. While public-health responses to risky sexual behaviour have often focused on health outcomes, in-depth qualitative research has indicated that social and economic outcomes are often valued higher than health outcomes (37). Research is increasingly drawing attention to the linkages among gender, sexuality, and poverty in the analysis of vulnerability to HIV and AIDS. Economic factors and imbalance in gender and power relations in sexual negotiations are major determinants of vulnerability of women to HIV infection (41). Oppong provided an in-depth analysis based on evidence from sub-Saharan Africa which links the vulnerability of women to HIV to the social construction of gender in the context of the economic, political, military and social crises which are transforming familial and social institutions: “There is no doubt that migration, urbanisation, education and the dislocation of customary forms of domestic organisation, based on traditional systems of kinship and marriage, are having profound effects upon the sexual behaviour of both women and men… [C]ertain population groups are already known to be particularly vulnerable and the epidemic has been categorised as increasingly affecting the youth and women, with proportions and numbers in these categories increasing rapidly and young women the most vulnerable of all… Instead of being enmeshed in lifelong systems of morally binding transactions of kinship and affinity, ensuring group solidarity and some measure of security across generations for young and old, individuals are forced more and more to rely upon precarious forms of livelihood in strange environments. They are drawn to engage in forms of short-term, unprotected, deregulated, opportunistic, economic and sexual behaviour which are entailed by such forms of survival…” (29).

A crucial aspect of vulnerability to HIV in the context of declining economic conditions is the breakdown of essential social support systems (38). The wealth of literature concerned with the reproductive health of young people shows that the risk of HIV infection for young people in developing countries is increased by sociocultural, political and economic forces, such as poverty, migration, war, and civil disturbance. Inequalities in age interface with inequalities in social and economic opportunities, gender, and sexuality to increase the vulnerability of young people to HIV and AIDS (16—for an extensive review of this literature).

As the AIDS pandemic has continued to expand, dissatisfaction with mainstream sexual behaviour research has increased. Large-scale surveys of knowledge, attitudes, practices and beliefs have increasingly given way to in-depth qualitative studies which examine sexual culture in terms of the social representations, symbols, and meanings that shape and structure sexual experience (40, 42). The preoccupation with epidemiological questions in sexual behaviour research, and the focus on the individual, has increasingly been replaced by an effort to identify the social, cultural, economic and political dimensions of sexual behaviour, drawing upon methods from the social sciences (43) and informed by Bronfenbrenner's social-ecological theory (44) and Marmot and Wilkinson's work on multilevel approaches to understanding social determinants of health (45). An understanding of the social contexts of sexual behaviour is a prerequisite for designing locally-appropriate HIV and AIDS interventions.

**A SOCIAL ANALYSIS OF REPRODUCTIVE HEALTH**

The framework for a social analysis of reproductive health presented below emphasizes the need to pay due attention to conceptual issues around context. Such a framework moves analysis from the macro to the local level and incorporates community perspectives, including those of the most vulnerable primary stakeholders*. A cautionary note is, however, needed. The framework as presented is comprehensive. No social analysis will be able to gather (or indeed interpret) information on all the issues contained within the framework. Nor should all the data to be collected be primary: much information is available from secondary sources. The framework should instead be viewed as a menu from which to select key issues and concepts according to not only the social and programmatic context, but to the resources (including funding, time, and expertise) available to the analyst.

### Social Context

An important starting point for any social analysis is the overall social and economic contexts in which reproductive health is experienced. The key elements in any such analysis are the nature and dynamics of poverty, human development, and social exclusion.

**Poverty analysis:** An analysis of poverty should start with a consideration of how poverty is defined in national sectoral policy statements, e.g. by per-capita income, distribution of income, access to resources. This should be followed by analysis of variations in poverty levels by geographical region, gender, ethnicity, seasonality, etc.; of how poverty is reflected in key health indicators, such as infant mortality and prevalence of HIV; and finally, which groups are the most disadvantaged or have the highest incidence of poverty, e.g. ethnic groups, rural women, agricultural labourers.

**Human development indicators:** The human development index (HDI), developed by the United Nations Development Programme, is an alternative to income-based measures of poverty in recognition that development is not simply a product of rising income levels. The HDI uses national indicators, such as life-expectancy, infant mortality rates, maternal mortality ratios, and access to basic education and basic welfare resources. In many countries, particularly in eastern and southern Africa, HIV/AIDS has been identified as the greatest threat to gains made in HDIs over the past few decades. A social analysis should include a discussion of human development indicators, including trends in national HDIs, disparities in HDIs (by region, gender, ethnicity), constraints on improvements in HDIs, and estimates of impact/potential impact of HIV/AIDS on HDIs.

**The dynamics of social exclusion:** Poverty is not simply economic, but related to vulnerability and social exclusion. Social exclusion, a central concept in social development policy, refers to the multi-dimensional character of deprivation: disadvantages resulting from gender, ethnic and age discrimination; lack of secure employment opportunities (including exclusion from employment-based social security); poor public-sector health and welfare services; and lack of access to mechanisms for participation. In this regard, social exclusion is closely linked to concepts of human development, vulnerability, and social capital. The study of social exclusion is not, however, a substitute for poverty analysis. While a focus on poverty—the structural aspects of deprivation, such as lack of food and housing, and their impact on individuals, households, and communities—remains central to social analysis (46), an analysis of social exclusion should focus on the processes, mechanisms, and institutions that exclude certain groups from accessing resources, assets, goods, and services. See Table 1 and the seminal work on health inequalities by Navarro and Muntaner (47).

**Table 1.** Analysis of the social dynamics of exclusion

Who are the marginalized and excluded groups? What factors contribute to their exclusion (e.g. gender, age, livelihood strategies, location, and social status)? An analysis of the social processes contributing to poor reproductive health among identified groups will include issues, such as:
• income deprivation, employment structures and processes, and labour migration
• urbanization and changes in social-support structures, e.g. changes in extended family structures and rise in female-headed households
• factors contributing to exclusion from access to productive assets and capital, e.g. gender relations and inheritance rights
• factors contributing to exclusion from access to reproductive health information and services, e.g. by age, marital status, ethnicity, gender, location, and disability

**The national policy and legislative framework:** In addition to a focus on the social and economic context, analysis at the macro-level should include the effects of the policy and legislative framework on the reproductive rights of vulnerable and excluded groups (Table 2).

**Table 2.** Social analysis of the policy and legislative framework

Equity in health-sector policy, for example:
• Which groups are reached by services, and which are excluded?
• Barriers to access to public and private-sector services, such as cost of services (user-fees/cost-sharing), and structural constraints to quality of care (provider training, referral systems, client-provider relations, supplies/logistics, location of service-delivery points)
• Are financing frameworks pro-poor? (e.g. do they favour poor regions?) (48)
Reproductive health policy and legislation on, for example:
• Provision of reproductive health information and services to young/unmarried people
• HIV/AIDS, e.g. level of recognition of the problem, general awareness-raising or targeted intervention approaches
• Distribution of condoms (free distribution? user fees?)
• Abortion, rape, sexual abuse, child abuse, etc.
Policy-level support to enabling environments, for example:
• Gender-based rights and entitlements reflected in national legal/policy frameworks?
• Are there sources of systemic, institutional bias against the needs of poor and socially-excluded groups? (gender, ethnic and class discrimination) (48)
• How does the policy environment support the rights of excluded groups to protect themselves from HIV/AIDS and poor reproductive health? Does the policy environment address the rights of people living with HIV/AIDS?

### The social dynamics of vulnerability

The social context of reproductive health needs to be understood from the perspective of the poorest, and the most marginalized and socially excluded. The concept of vulnerability moves beyond notions of individual risk behaviour to a consideration of the contexts in which people are placed at risk of poor reproductive health outcomes. While vulnerability is linked to poverty, poor reproductive health may not be the effect of poverty alone, but to processes and forms of power which lead to social exclusion. An underlying determinant of social vulnerability is the extent to which people are able to realize their rights to protection from risk and to access services and resources. The six key issues in the analysis of social vulnerability are:

**Poverty, livelihood strategies, and social capital:** The economic causes of vulnerability to poor reproductive health, and how poverty impacts on the health, sexuality, and fertility of primary stakeholders, are central concerns (Table 3).

**Table 3.** Poverty, livelihood strategies, and social capital

Livelihood strategies: How do livelihood strategies impact on reproductive health, for example, in relation to:
• patterns of sexual networking (economically-based sexual exchange and commercial sex)
• power dynamics in negotiating sexual relationships, e.g. safer sex/condom-use
• the economic value of children (the role of children in livelihood strategies)
• specific groups, e.g. migrant workers, female household heads, young people, women engaged in commercial sex and their partners
Household access to economic resources: What differences are there in levels of household access to economic resources? How does intrahousehold access to economic resources and decision-making impact on access of different household members, e.g. by gender/age, to food, healthcare (including importance given to maintenance of good health, e.g. preferences of male child)
Social capital is closely linked to livelihood strategies and refers to both social resources, such as kin groups, community organizations, peer networks, and symbolic assets, such as ancestral lineages, spiritual resources, church affiliation, on which different groups draw as a means of social security. What sources of social capital do people draw on? How do sources of social capital impact on health, sexuality, and fertility, for example:
• social and symbolic value of sexual relationships
• symbolic value of children, e.g. for maintenance of the lineage
• value of spiritual advisers and their impact on health-seeking behaviour
• church affiliation and teachings concerning sexuality and reproduction

**Gender and vulnerability:** Reproductive health is not only culturally-specific, but also gender-specific. Decisions relating to sexuality, fertility, reproduction, and health may be determined by a range of gender-specific factors, such as relations of power and control within marriage, households, and kin groups; the economic and symbolic value of fertility; women's position regarding paid work and access to childcare resources (Table 4).

**Table 4.** Gender analysis in local context

Consider how gender identities, and gender roles and responsibilities, are constructed in the local context, for example:
• how gender-based rights and entitlements are defined within marriage, the household, and the lineage/extended kin group?
• how gender identities and power relations intersect with other power relations and identities, such as class, ethnicity, church/religious affiliation, sexuality, and age?
• gender dynamics of participation in formal and informal decision-making structures
• gender-based access to formal and informal social networks and social support
• gender-based access to economic resources and services
• gender dynamics of economic survival strategies among the poorest
• impact of gender identities and relations of power and control on the vulnerability of different groups to poor reproductive health outcomes
• diversity of gender-based reproductive health needs among primary stakeholders, e.g. according to livelihood, life-cycle position, ethnicity, etc.

**Local knowledge and health-seeking behaviour:** Reproductive health programming requires an awareness of local systems of knowledge and health practice (49). Cultural context is central to the response to health and illness, with knowledge relating to health and ill-health shaped by culturally-specific practices which vary between different social groups or networks. Consequently, the meanings attributed to health-related behaviour by health professionals are often very different to those of lay-people. The social experience of health and illness contributes to the construction of local knowledge that informs health-seeking behaviour (Table 5) (50, 51).

**Table 5.** Local knowledge and health-seeking behaviour

Analyze the key issues relating to experiences of health and ill-health and the resources (social capital) available to maintain health and alleviate suffering as a result of ill-health, and the decision-making process involved in seeking treatment options, such as:
• local understandings and definitions of health and ill-health
• sources of knowledge on which people draw to explain causes of common illnesses and poor health, e.g. local belief and knowledge systems, including categories of common illnesses, spiritual/religious knowledge, biomedical knowledge
• sources of healthcare available to different groups, e.g. categories of traditional healers, government services, private providers, community agents, pharmacists, informal support networks, kin, and mothers/mothers-in-law
• sources of social support on which people draw in the event of ill-health
• patterns of health-seeking behaviour among different groups as they relate to experiences of different illnesses, e.g. STIs, HIV/AIDS, maternal health, infant and child health
• relationship between poverty, social identity (social capital and exclusion), and health-seeking behaviour and choice of healthcare provider

**Sexuality, sexual behaviour, and sexual health:** The social context in which sexual behaviour takes place encompasses a range of institutions and relations (family relations, gender relations, economic relations, religion, ethnicity, mobility) and meanings people attribute to sexual relations (Table 6).

**Table 6.** Sexuality, sexual behaviour, and sexual health

How are sexuality and sexual behaviour understood and experienced locally? Key issues might include:
• the diversity of sexual behaviour among different groups of primary stakeholders
• sources of knowledge on sexuality and sexual health, e.g. traditional mechanisms for sex education, kinship relations, and impact of urbanization/modernization/media
• how different sexual identities and relationships are constructed and experienced, e.g. commercial sex, same sex relationships, boyfriend/girlfriend relationships, and marital/extra-marital relationships
• patterns of sexual networking and exchange among different primary stakeholders, and how these link to gender and power relations
• how poverty/livelihood strategies impact on sexual behaviour and vulnerability of different groups
• aspects of the social construction of sexuality, gender, power, and economic relations which increase the vulnerability of particular groups to poor sexual health outcomes
• sources of support/advice on which different groups draw if they have sexual health concerns

**Reproduction and fertility:** Reproductive behaviour and decision-making are embedded in social relations and institutions that operate from the macro to the micro level (kinship groups, gender relations and culturally-defined gender roles, economic and labour relations, traditional health systems, local political structures, religious affiliations, and informal peer networks) (Table 7).

**Table 7.** Reproduction and fertility

Consider the meanings given to fertility and reproduction locally, including analysis of how local reproductive strategies are linked to other aspects of social identity and organization, such as class, gender, ethnicity, power relations, kinship structures, religion, and local health and belief systems. Issues to consider include:
• social and economic value given to children by different groups
• networks/resources/social support on which different groups draw for childcare
• sources of reproductive knowledge on which different groups draw
• social value and meanings given to fertility and infertility
• fertility decision-making processes and networks (who exercises power and control: individual women, husbands, reproductive couples, kin groups, mothers-in-law?)
• fertility-control practices and sources of fertility control (traditional methods and modern contraceptive services)
• perceptions of different fertility-control methods and their accessibility, acceptability, and appropriateness to different groups of primary stakeholders
• local reproductive practices and preferences (birthing practices, postnatal care [care of the newborn and care of the placenta], and maternal healthcare, etc.)

**Access to quality services:** Access and quality are inter-related (Table 8) (52). If demand represents the social context in which health, sexuality, and reproduction are experienced and acted upon, access is the social interface between services and the community. Assessing access requires determining the extent to which services may be obtained at a level of effort, and of monetary, opportunity and social cost, that are acceptable to and within the means of poor, marginalized and vulnerable people. A range of factors determines access: availability, affordability, acceptability, convenience, and knowledge. Quality is the social experience of services and relates to the extent to whether a programme is responding to the perceived needs and demands of clients and potential clients. Assumptions of clients and potential clients about quality often differ from those of providers, managers, and policy-makers. A critical but often neglected aspect of quality is client-provider interactions, an understanding of which is essential for improving the quality of care.

**Table 8.** Access to quality services

Access to information
• Where/how do different groups access information on STIs/HIV/AIDS, family planning, child health, maternal health, etc., and on the services available to them?
• What are the most culturally-appropriate and accessible sources of information (from the perspective of primary stakeholders)?
• What are the information needs among different groups of primary stakeholders?
Social access to services
• What services are available to different groups in the community?
• Are services accessible to different groups (culturally, geographically)?
• Which services/providers do different groups feel most comfortable using?
• What are the important factors influencing health-seeking behaviour and decision-making regarding the use of providers and services?
• What are the key barriers to access identified by different primary stakeholders?
• How appropriate is the current service-delivery system to locally-identified needs?
Economic access to services
• How does access to income impact on access to health services?
• What costs are incurred by primary stakeholders in using different services (user-fees, travel costs, cost of drugs/prescription charges)?
• Which services are not affordable to primary stakeholders? Which groups are excluded from accessing services because of cost?
• Do the poor have equal access to good-quality services?
Quality of care
• Community/primary stakeholders' perceptions of:
- communications and relationships with different service providers
- technical competence and skills of different service providers
- appropriateness and effectiveness of different treatments/services (treatment of STIs, family-planning methods, antenatal and postnatal care, etc.)
- problems with treatment/products (side-effects, etc.)
• Are service providers responsive to meeting needs of the poor and socially excluded?
• Are the reproductive health needs of different groups not currently being met?

## CONCLUSION

### Interventions and strategies

One of the key outcomes of an analysis such as that outlined above will be a set of interventions and strategies. The absence of detailed and systematic social analyses of reproductive health leads in many cases to such interventions and strategies being somewhat formulaic and not tailored to the specific local context. We provide here four examples of the kinds of programmatic and policy responses indicated by a detailed social analysis:

### Addressing reproductive rights

The rights-based approach to development is a core concept in current social policy discourse. As noted, the rights discourse runs the risk of focusing on the rights of the individual to the exclusion of the wider context within which rights are defined and realized. Reproductive health depends upon the extent to which poor and marginalized groups are able to realize their rights to economic and social resources. Creating and/or supporting social, political and physical environments that enable the poor and marginalized groups to realize their rights to access resources can provide an important basis for poverty elimination: “…a concern for economic growth needs to be matched by attention to ensuring equitable distribution and access to public resources, and safeguards to protect the interests of the poor and vulnerable” (53). The identification of causes of poverty, social marginalization, and social exclusion is, therefore, essential in any social analysis. People are unable to exercise their rights if their livelihoods are endangered, public-health and education systems are inadequate, and cultural diversity and ethnic identity are not respected (54).

Key elements of the rights-based approaches in reproductive health programmes include equity-based objectives which focus upon the poor and socially-excluded, implementation, and evaluation with the full participation of intended beneficiaries, priority attention to poverty elimination (including the rights to safer livelihood strategies to ensure adequate standards of living, access to food/housing, access to education, and health), and the development and use of indicators for measuring progress in improving the quality of life of the poor and marginalized.

Approaches which address the rights of vulnerable groups will include working with the government to create a policy environment to protect their rights (e.g. protection from violence and sexual abuse); strengthening civil society groups, including women's rights networks; working with power-brokers, such as police, to reduce abuse of rights of vulnerable groups, such as commercial sex workers; and legal support for protection of the rights of vulnerable groups.

### Conditions to support behaviour change

The realization of the rights of vulnerable groups to protection from risk and from poor reproductive health outcomes is dependent upon conditions being in place which support behaviour change. Creating the supportive environments for behaviour change at the local level may encompass a range of elements, such as:

**Peer support networks:** The most effective communication approaches are those in which behaviour change is reinforced from within peer groups and in which information is received and exchanged based on relationships of trust. Enabling environments need to be created to support and motivate peer educators and community agents (e.g. through training, supervision, and incentives).

**Enhancing existing sources of social capital:** Social capital refers to social institutions, social networks, and social relationships that shape the quality of a society's social interactions: “Social capital is not just the sum of institutions which underpin a society, but the glue that holds them together” (55). Capacity-building of the existing informal and formal community-based support networks and organizations is essential for enhancing social capital and for bringing about sustained behaviour change. Capacity-building of community structures includes support to self-help groups/community-based care/support networks (e.g. for people living with HIV/AIDS), support to microcredit schemes to reduce economic vulnerability and insecurity, strengthening rights and health networks of women and informal and formal networks of men to address issues of gender equity and reproductive health, and strengthening existing mechanisms for community participation, e.g. village-development committees, women's groups, management of community health, and the capacity of community-based groups to undertake advocacy work.

### Increasing access of vulnerable groups to resources and services

Approaches to increasing access for marginalized and vulnerable groups to healthcare and reproductive health services need to be based on an understanding of health-seeking behaviour and the needs of primary stakeholders. Strategies for increasing access should focus on improving the quality of public-sector services, developing/strengthening community-based health outreach and delivery systems and referral networks between community-based and other service-delivery structures, integration of reproductive health into existing primary healthcare networks and clinic services, training of non-state service providers, such as traditional birth attendants, private providers, pharmacists, and other sales agents, in minimum standards to improve the quality of care, and the development of innovative and alternative community-based condom-distribution systems, including free peer-to-peer distribution, condom social marketing, and distribution through outlets, such as bars, night-clubs and brothels.

### Responsiveness of services to client needs

Building mechanisms into programmes to allow upward accountability in policy, institutional and service-delivery systems is essential to improve the responsiveness of services to the local realities of users and potential users. A crucial element of systems of accountability is the monitoring of interventions/services from the community perspective to ensure: equitable access for the poor and most vulnerable to prevention, treatment, and care; gender equity in accessing health resources, and prevention and care services; reduced regulatory constraints on the marginalized and vulnerable accessing services; access to reproductive health information and education; appropriate choice of services to meet needs; informed client demand for services; client satisfaction regarding interactions with providers, including provider skills and technical competencies; client satisfaction with availability and continuity of supplies and products and with follow-up and referral arrangements; and that services meet the needs of clients and local expectations of quality of care.

### Summary

Good reproductive health outcomes mean different things to different groups, in different social contexts, and hence, programmes and policies must take account of actors' perspectives. The challenge is to find an approach to policy and programme development that is both true to the experience of marginalized and excluded groups, and which is grounded in an understanding of social conditions of vulnerability. The framework for social analysis outlined here aims to facilitate such an approach. The framework also has a direct bearing on policy analysis and policy formulation. The view that there is one objective version of reality (in this case reproductive behaviour) which can be captured and explained through individualistic and normative paradigms has been challenged. Rather, reproductive health programming is a messy and complex business, in which social actors (the so-called programme beneficiaries) are continually trying to develop and negotiate strategies for dealing with competing interests and multiple perspectives in different social situations.

## ACKNOWLEDGEMENTS

We acknowledge the funding received from the Department for International Development, UK, for the research on which this article is based. However, the opinions expressed in this article are those of the authors alone, and the authors accept full responsibility for any errors or shortcomings.

## Footnotes

*The marginal are those individuals and groups who are excluded from the economic and social resources of mainstream society. They may be defined as living on the edge of society: socially excluded and/or socially liminal. Examples of the socially excluded include people living with HIV and AIDS, ethnic minorities, etc; socially liminal include adolescents and unmarried adult women, including widows and divorcees, in societies where marriage is universal.

*Incorporating the views of the most vulnerable or marginal stakeholders is challenging. It is not within the scope of this article to set out detailed practical guidelines for how to do this, but the reader is directed to the vast literature on participation (56)
